# Direct observation of ultrafast coherent exciton dynamics in helical π-stacks of self-assembled perylene bisimides

**DOI:** 10.1038/ncomms9646

**Published:** 2015-10-23

**Authors:** Jooyoung Sung, Pyosang Kim, Benjamin Fimmel, Frank Würthner, Dongho Kim

**Affiliations:** 1 Spectroscopy Laboratory for Functional π-electronic Systems and Department of Chemistry, Yonsei University, 134 Shinchon-dong, Seodaemun-gu, Seoul 120-749, Korea; 2 Institut für Organische Chemie and Center for Nanosystems Chemistry, Universität Würzburg, Am Hubland, Würzburg 97074, Germany

## Abstract

Ever since the discovery of dye self-assemblies in nature, there have been tremendous efforts to exploit biomimetic supramolecular assemblies for tailored artificial photon processing materials. This feature necessarily has resulted in an increasing demand for understanding exciton dynamics in the dye self-assemblies. In a sharp contrast with J-type aggregates, however, the detailed observation of exciton dynamics in H-type aggregates has remained challenging. In this study, as we succeed in measuring transient fluorescence from Frenkel state of π-stacked perylene tetracarboxylic acid bisimide dimer and oligomer aggregates, we present an experimental demonstration on Frenkel exciton dynamics of archetypal columnar π–π stacks of dyes. The analysis of the vibronic peak ratio of the transient fluorescence spectra reveals that unlike the simple π-stacked dimer, the photoexcitation energy in the columnar π-stacked oligomer aggregates is initially delocalized over at least three molecular units and moves coherently along the chain in tens of femtoseconds, preceding excimer formation process.

## Abstract (web summary)

Molecular self-assemblies have potential as photon processing materials, yet observation of exciton delocalization dynamics can be challenging. Here, the authors experimentally demonstrate Frenkel exciton dynamics of H-type aggregates, studying delocalisation of excitons directly after photoexcitation.

## Graphical abstract

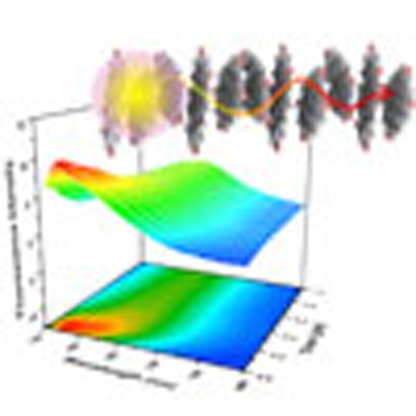

The discovery of molecular dye self-assemblies in nature such as light-harvesting complexes in plants and several kinds of bacteria and algae has captured the imagination of many researchers, inspiring numerous works to fabricate functional molecular dye aggregates^1^,^2^,^3^. Moreover, advances in supramolecular chemistry and molecular nano-engineering have paved the way to devise tailor-made artificial photon processing materials for the future applications of molecular assemblies^4^,^5^,^6^. The fact that the performances of the electronics and photonics depend strongly on the optical properties of these materials has driven the need for a more fundamental understanding of exciton transport in molecular assemblies^7^,^8^.

Numerous researches have shown that the photophysics of molecular aggregates can be understood using the idea developed decades ago by Kasha and co-workers^9^,^10^. According to the Frenkel exciton model, the electronic excitation in molecular aggregates spreads over many monomers in the form of excitation waves^2^. Like quantum coherence in photosynthetic systems, this exciton delocalization was suggested to enhance exciton transport in the artificial molecular aggregates^11^,^12^. On the other hand, dynamical localization, produced by coupling to internal and external vibrational modes, imperfections of aggregates and disorder induced by environment, restricts the size of the exciton wave function and hence modifies the optical and transport properties of excitons^13^. When excitation becomes more or less localized on one monomer, the excitation energy transfer is no longer described by coherent exciton motion but it is generally assumed to be an incoherent hopping process^12^,^14^. In other words, the degree of exciton delocalization determines exciton behaviour (that is, coherent wave-like or incoherent hopping motion) and, therefore, the rate of exciton migration. Accordingly, it is crucial to understand exciton delocalization and localization dynamics in molecular aggregates for ensuring high device efficiencies.

Among numerous molecular aggregates, sandwich-type π–π stacked dye assemblies, namely H-aggregates, have attracted considerable attention, since they can serve as constituent materials for electronic and optoelectronic devices, such as bulk heterojunction solar cells, field effect transistors and organic light emitting diodes^15^,^16^,^17^,^18^. Unfortunately, the small oscillator strength of the lowest Frenkel state in H-aggregates and its weak fluorescing nature render detailed observation of the dynamics very challenging^19^. In particular, the line shape changes in the excited-state absorption and stimulated emission spectra of the exciton state are overwhelmed by spectral congestion in femtosecond-transient absorption measurements. Therefore, despite numerous researches, it has long been challenging to understand the exciton migration behaviour of the primarily generated exciton in π–π stacked self-assemblies of dye molecules. Nevertheless, recent theoretical research has suggested fluorescence measurements as an attractive complementary method to determine exciton delocalization size^20^. Since the degree of electronic correlation between delocalized molecular sites is reflected in vibronic coupling, the dynamic information about exciton delocalization size can be deduced by tracking down the 0-0 to 0-1 vibronic peak ratio in the transient fluorescence spectra^8^,^20^,^21^,^22^.

In this study, we have focused on observing emission from the lower Frenkel exciton state of an archetype H-type aggregate so as to analyse the exciton delocalization dynamics. As representative systems for H-type aggregates with face-to-face stacking geometry, we have prepared two perylene tetracarboxylic acid bisimide (PBI) aggregates PBI **1** and PBI **2**, which are distinguished by their size, that is, dimer for PBI **1** and extended oligomer for PBI **2** (Fig. 1a,b)^23^,^24^,^25^,^26^. To the best of our knowledge, except emission from relaxed excimer state of such aggregates, the observation of emission from the initial Frenkel state of any H-aggregates including these PBI aggregates has never been reported. Thus, although the exciton relaxation processes and the role of excited species of H-aggregates have been extensively investigated, the exciton dynamics, in particular, the delocalization and localization processes of primarily generated exciton have remained unexplored. Here we present, for the first time, time-resolved fluorescence study of π–π stacked self-assemblies obtained by a broadband fluorescence upconversion technique. The fruitful information on interaction-induced change in vibronic line shapes of the fluorescence spectra disentangles the intrinsic dynamics of the delocalized exciton. Furthermore, a comparative study on PBI dimer and the extended PBI aggregates allows us to estimate exciton localization, a process of relevance in light-harvesting systems of photosynthesis and organic electronic devices.

## Results

### Exciton coupling strength

**Figure 1:**
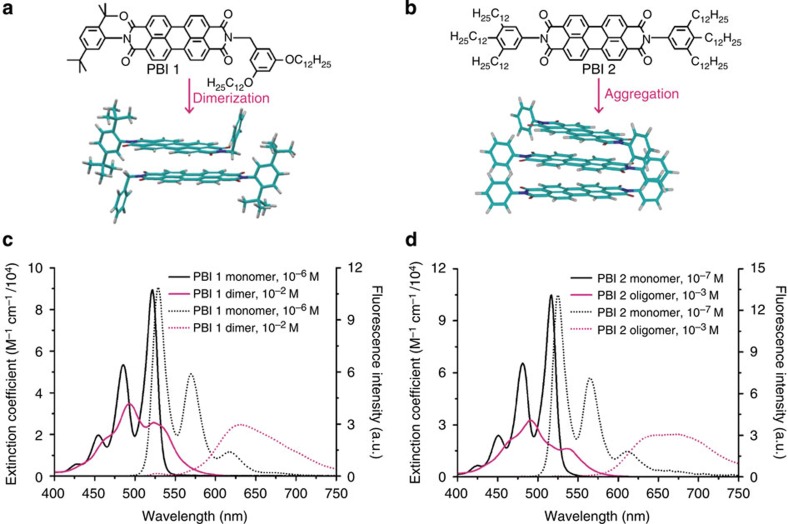
Schematic diagram of self-assembled geometries and steady-state absorption and emission spectra of PBI 1 and 2. Molecular structures and the stacking model of PBI **1** (**a**) and PBI **2** (**b**). (**c**) steady-state absorption (solid line) and emission spectra (dotted line) of PBI **1** monomer (1.0 × 10^−6^ M, black line) and PBI **1** dimer aggregate (1.0 × 10^−2^ M, pink line) in a solvent mixture of CHCl_3_/MCH. (**d**) steady-state absorption (solid line) and emission spectra (dotted line) of PBI **2** monomer (1.0 × 10^−7^ M, black line) and PBI **2** oligomer aggregate (1.0 × 10^−3^ M, pink line) in MCH.

We begin by reviewing the salient features of the absorption spectra of PBI **1** dimer and PBI **2** oligomer aggregates. The absorption spectra of PBI **1** were measured in the solvent mixture of CHCl_3_/MCH (v:v=1:5; methylcyclohexane (MCH)) at above 10^−2^ M, where >80% of PBI **1** exists as π-stacked dimers, while those of PBI **2** were recorded in pure MCH at above 10^−3^ M, where almost all PBI **2** molecules are embedded in π-stacks that are composed of at least 10 monomers^24^,^26^. Besides, previous MM3* geometry optimization study of PBI **1** dimer and molecular modelling studies of PBI **2** oligomer aggregates revealed that both PBI aggregates form very similar intermolecular geometry, being stacked in a helical manner with the intermolecular angles of 26° for PBI **1** dimer and 30° for PBI **2** oligomer aggregates (Fig. 1a,b)^25^,^26^,^27^. As shown in Fig. 1c,d, the absorption spectra of PBI **1** and **2** monomers exhibit pronounced vibronic progressions, resulting from coupling between the main S_0_–S_1_ electronic transition and the symmetric vinyl stretching mode with frequencies near 1,400 cm^−1^ (refs 28, 29). On the other hand, PBI **1** dimer and PBI **2** oligomer aggregates show characteristic aggregation-induced shifts in the absorption spectra, indicative of excitonic interactions between the PBI subunits^28^,^29^,^30^.

According to the excitonic coupling theory, H-aggregates lead to positive excitonic coupling and the placement of the allowed exciton state at the top of the exciton band exclusively^8^,^21^,^22^. Although rotational displacement between the neighbouring subunits of PBI **1** dimer and PBI **2** oligomer aggregates weakens the selection rule and provides some probability for the Frenkel exciton to reside at the antisymmetric forbidden bottom band, the large exciton band intensity ratio of symmetric to antisymmetric components indicates that the main contribution to absorption spectrum stems from the transition to the symmetric upper band^22^,^30^,^31^. Thus, we can evaluate exciton coupling strength, *J*, by performing the systematic analysis of the effect of excitonic interaction on the vibronic line shapes (see Supplementary Note 1). As shown in Supplementary Figs 1–3, the *J*-dependent absorption spectra of PBI **1** dimer and PBI **2** oligomer aggregates were reproduced from the well-established theoretical model for vibronic band ratio of molecular aggregates^30^,^31^,^32^. By comparison of the simulated absorption spectra with the measured absorption spectra, we have estimated the exciton coupling strength of ∼500 cm^−1^ for PBI **1** dimer and ∼700 cm^−1^ for PBI **2** oligomer aggregates, respectively, commensurate with our previous quantum chemical studies^31^. It is noteworthy that despite different number of stacking units, both PBI **1** dimer and PBI **2** oligomer aggregates have intermediate coupling strength, where the vibrational energy, *ω*_0_, the vibronic relaxation energy, *λ*^2^*ω*_0_, and the free exciton bandwidth, *W*, are all comparable in magnitude^8^.

We now consider the steady-state emission spectra of PBI **1** dimer and PBI **2** oligomer aggregates as shown in Fig. 1c,d. While each of the PBI **1** and **2** monomers exhibits a vibronically well-resolved emission spectrum, the mirror image of the absorption spectrum, each of the PBI **1** dimer and PBI **2** oligomer aggregates shows a broad and much red-shifted emission spectrum, which is far from the mirror image of the absorption spectrum. When excitonic coupling is present, the excitonic state splits into two separate higher energy symmetric (top band) and lower energy antisymmetric excitonic states (bottom band) resulting in the two peaks, so-called Davydov components^32^. In accordance with Kasha's rule^33^,^34^, emission comes from the lowest excited state, which is the antisymmetric state for PBI **1** dimer and PBI **2** oligomer aggregates. For columnar dye stacks optical transitions between the ground and this antisymmetric state are not perfectly forbidden due to a rotational offset between the aggregated dyes. The angles between the transition dipoles of two neighbouring subunits of PBI **1** dimer and PBI **2** oligomer aggregates are 26 and 30°, respectively, in the ground and Franck–Condon excited states^35^. In addition, due to the intermediate coupling strengths of PBI **1** dimer and PBI **2** oligomer aggregates, the thermally activated symmetric upper band could enhance the 0-0 emission band^8^,^20^. Thus, in spite of stacking arrangement with predominant H-type coupling, we expect non-negligible 0-0 emission band from the bottom exciton state. However, as aforementioned, the steady-state emission spectra of PBI **1** dimer and PBI **2** oligomer aggregates appear in the far more red-shifted range of 600–750 nm with broadened shape. This large difference in spectral position between the low-lying absorbing state and the experimentally observed emitting state indicates fast excited-state dynamics of PBI **1** dimer and PBI **2** oligomer aggregates in the interim.

### Emission from Frenkel exciton state

**Figure 2:**
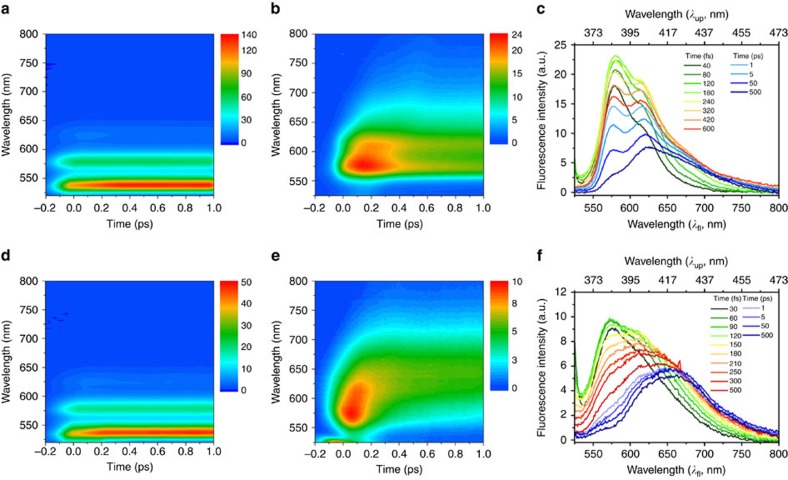
2D fluorescence maps and transient fluorescence spectra of PBI 1 dimer and PBI 2 oligomer aggregates systems. (**a**) Two dimensional (2D) fluorescence map of PBI **1** monomer in CHCl_3_. (**b**,**c**) 2D fluorescence map and transient fluorescence spectra of PBI **1** dimer aggregate in a solvent mixture of CHCl_3_/MCH. (**d**) 2D fluorescence map of PBI **2** monomer in CH_2_Cl_2_. (**e**,**f**) 2D fluorescence map and transient fluorescence spectra of PBI **2** oligomer aggregate in MCH. All the spectra were obtained by femtosecond broadband fluorescence upconversion technique. Although the excitation wavelength was set to 495 nm to avoid strong spectral interference of the pump pulse, the sum-frequency signals generated between pump and gate pulse were observed in wavelength range over 550 nm.

To directly track down the exciton dynamics of PBI **1** dimer and PBI **2** oligomer aggregates, we have utilized broadband fluorescence upconversion technique. Time-resolved fluorescence spectra were acquired after photoexcitation at 495 nm so as to avoid spectral interference caused by the strong pump pulse. As shown in Fig. 2a,d and Supplementary Figs 4 and 5, the time-resolved fluorescence spectra of PBI **1** and **2** monomers do not show significant spectral evolutions in the picosecond time range. In contrast, PBI **1** dimer and PBI **2** oligomer aggregates reveal interesting spectral features in the time-resolved fluorescence spectra (Fig. 2b,c,e,f and Supplementary Figs 6 and 7). In particular, the early-time fluorescence spectra appear in 530–750 nm with dominant vibronic peaks at 580 and 618 nm for PBI **1** dimer and 575 and 615 nm for PBI **2** oligomer aggregates, which are clearly distinct from its respective steady-state fluorescence spectrum. The spectral position of the first vibronic band exists in the proximity to that of the lower energy antisymmetric excitonic state. These spectral features are often observed in J-aggregates, which are stacked in head-to-tail manner, indicative of an initial delocalized Frenkel exciton^19^,^36^,^37^,^38^. However, until now, for planar π-conjugated molecules having a face-to-face arrangement with predominant H-type coupling, only red-shifted bands without vibronic features in the vicinity of the bottom emission band have been observed. Thus, this observation provides the first direct experimental evidence on the initial formation of a Frenkel exciton in H-aggregates of planar π-conjugated molecules on photoexcitation. The initial time-resolved fluorescence spectra of both PBI **1** dimer and PBI **2** oligomer aggregates evolve within the first 1 ps. Afterwards, vibronic features disappear and the time-resolved fluorescence spectra become broad and red-shifted, being similar to the steady-state emission spectra of PBI **1** dimer and PBI **2** oligomer aggregates, respectively. In accordance with previous studies, we attribute the observed broad emission to excimer formation^23^,^26^,^39^. Consequently, we suggest a two-step mechanism where the formation of the excimer state is preceded by the Frenkel exciton state.

### Coherent exciton dynamics

To get a deeper insight into the coherent nature of Frenkel exciton and its behaviour in molecular aggregate systems, we have scrutinized the time-resolved fluorescence spectra of PBI **1** dimer and PBI **2** oligomer aggregates. In general, conjugated molecules exhibit well-resolved progressions involving the symmetric vinyl stretching mode. On aggregation, the degree of electronic correlation between molecular sites underlying a delocalized excited state changes so that the coupling of electronic excitation to vibration could be altered. As a result, this leads to the distortion of vibronic progression in the fluorescence spectrum. Accordingly, any electronic perturbation caused by aggregation is reflected in its fluorescence spectrum. Especially, the 0-0 peak depends entirely on the coherence of the emitting exciton, in a marked contrast with the mainly incoherent origin of the vibronic sidebands^40^,^41^. It has been proposed that the *I*^0-0^/*I*^0-1^ vibronic peak ratio in the transient fluorescence spectra provides a direct measure of the exciton coherence (or delocalization) size^20^,^21^,^22^. As we have succeeded in observing transient emission from the weakly allowed lowest Frenkel exciton state, it is now possible to evaluate the delocalization size in time.

It is obvious that the 0-0 peaks of PBI **1** dimer and PBI **2** oligomer aggregates decay rapidly within a few picoseconds. Meanwhile, in a relative sense, the 0-1 peak intensities seem to remain rather unchanged (Supplementary Fig. 8), which gives a hint on the coherent behaviours of the Frenkel exciton in molecular aggregates. For a deeper understanding of the coherent features in PBI **1** dimer and PBI **2** oligomer aggregates, we have performed a spectral analysis of the transient fluorescence spectra according to the method theoretically developed by Spano and co-workers^19^,^20^,^21^. In this approach the coherence size is reproduced by the 0-0 to 0-1 peak ratio multiplied by the Huang–Rhys factor (see Supplementary Note 2), which is^20^,^21^,


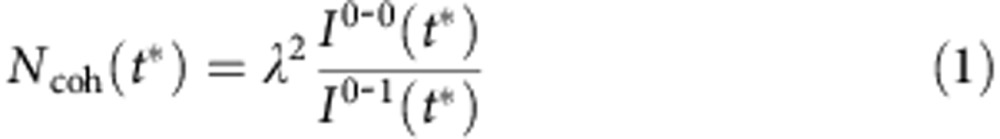


By integrating each vibronic peak (a detailed fit analysis is described in Supplementary Note 3 and Supplementary Figs 9 and 10), we could obtain the *I*^0-0^/*I*^0-1^ peak ratio and hence the exciton coherence size. It is noteworthy that owing to the incoherent nature of subside bands, the relative ratio of the 0-1 band to any higher order vibronic bands should be constant. However, because of ill-resolved transient fluorescence spectra and the appearance of excimer emission, it is hardly possible to differentiate the higher order vibronic progression in the transient fluorescence spectra of PBI **1** dimer and PBI **2** oligomer aggregates. Therefore, we roughly assigned all the higher vibronic progression bands, including excimer band, as *I*^0-*ν′*^_(*ν′*>1)_ and assume that any changes in the *I*^0-*ν′*^_(*ν′*>1)_/*I*^0-1^ peak ratio represent the excimer formation process.

## Discussion

**Figure 3:**
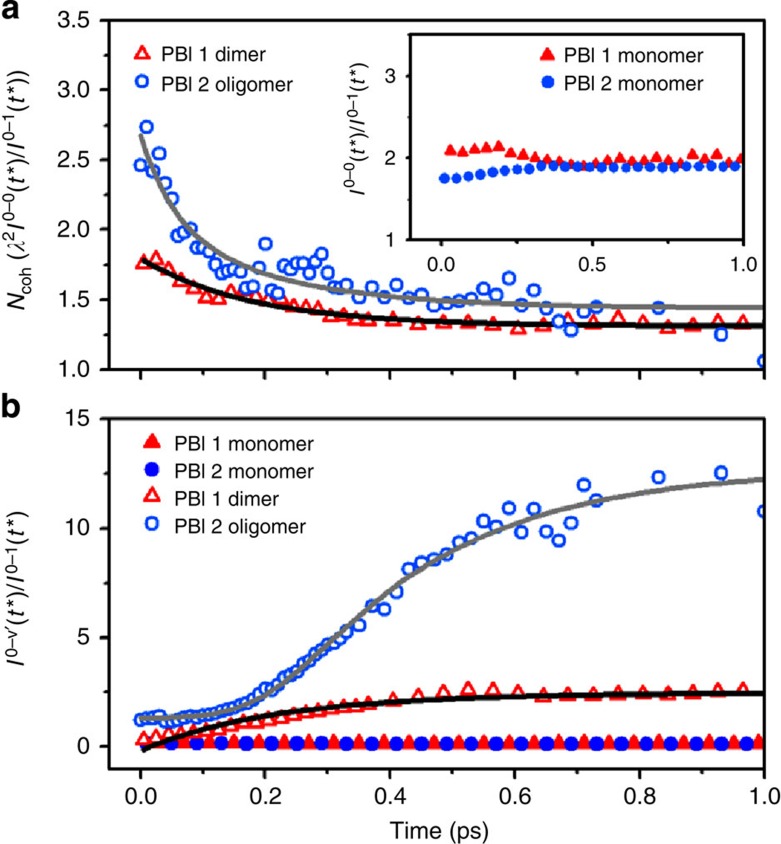
The vibronic peak ratio revealing coherence evolution in molecular aggregates. (**a**) The coherence lengths of PBI **1** dimer (red hollow triangle) and PBI **2** oligomer aggregates (blue hollow circle) as a function of time t*. Inset shows the *I*^0-0^/*I*^0-1^ vibronic peak ratio of PBI **1** monomer (red triangle) and PBI **2** monomer (blue circle) as a function of time t*. (**b**) the *I*^0-*ν′*^/*I*^0-1^ vibronic peak ratio of PBI **1** monomer (red triangle) and PBI **2** monomer (blue circle), PBI **1** dimer (red hollow triangle) and PBI **2** oligomer aggregates (blue hollow circle) as a function of time t*. All the data were obtained by analysing each transient fluorescence spectrum. The details of the analysis are given in Supplementary Note 3. See Supplementary Tables 1 and 2 for the fitting parameters.

As shown in Fig. 3a,b, the *I*^0-0^/*I*^0-1^ and *I*^0-*ν′*^_(*ν′*>1)_/*I*^0-1^ peak ratios in the transient fluorescence spectra of PBI **1** and **2** monomers remain constant, reflecting a lack of coherence in PBI monomers. On the other hand, the *I*^0-0^/*I*^0-1^ peak ratio of PBI **1** dimer decays with the time constant of 200 fs with the initial *N*_coh_ value of about 2 and gradually decreases within the next several picoseconds. This implies that the photoexcitation energy of PBI **1** dimer is coherently delocalized over a whole dimer entity but the coherence is suppressed rapidly within several hundreds of femtoseconds. As time evolves, the relaxation towards antisymmetric band bottom, induced by nonadiabatic coupling, leads to further quenching of 0-0 emission band and hence additional decrease in the *N*_coh_ (Supplementary Fig. 9)^20^. Along with the initial changes in the *N*_coh_, the *I*^0-*ν′*^_(*ν′*>1)_/*I*^0-1^ peak ratio starts to increase from the incipient state with the time constant of 220 fs. Since the *I*^0-*ν′*^_(*ν′*>1)_/*I*^0-1^ peak ratio describes the excimer formation process and its rise time scale is coincident with the decay of *N*_coh_, we can infer that the excimer formation process between the two neighbouring PBI units causes the coherent suppression of initially delocalized Frenkel exciton. It is noteworthy that the observed excimer formation processes in self-assembled PBI dimers are faster than those reported in earlier studies on covalent PBI dimers (that is, within a few hundreds of femtoseconds to a few tens of picoseconds)^39^. Thus we can attribute the ultrafast excimer formation process to the mixing between Frenkel and charge-transfer exciton states, which gears up for ultrafast exciton transfer to excimer state^42^,^43^,^44^,^45^.

Interestingly, in the case of PBI **2** oligomer aggregates, the trace of *N*_coh_ exhibits double exponential decay components with the time constants of <80 and 250 fs. Referring to the initial *N*_coh_ value of about 3 and the limited time-resolution of our broadband fluorescence upconversion set-up, the spatially coherent exciton should be initially delocalized over more than three subunits in the PBI **2** oligomer aggregates. The decay profile of *N*_coh_ then decreases to the value of 2 with the time constant of <80 fs. Continuously, the *N*_coh_ of Frenkel exciton state further drops within the next several hundreds of femtoseconds. On the other hand, the *I*^0-*ν′*^_(*ν′*>1)_/*I*^0-1^ peak ratio seems to be constant within the first 110 fs. Soon after, the *I*^0-*ν′*^_(*ν′*>1)_/*I*^0-1^ peak ratio starts to increase with a rise component of 255 fs. As observed in PBI **1** dimer, the excimer formation process and the concomitant decoherence of Frenkel excion in PBI **2** oligomer aggregates also occur on the similar time scale of about 200 fs.

The intriguing and significantly distinctive features of PBI **2** oligomer aggregates are given by an additional fast decay component of the *N*_coh_ and the invariant *I*^0-*ν′*^_(*ν′*>1)_/*I*^0-1^ peak ratio within the first tens of femtoseconds. As aforementioned, both PBI **1** dimer and PBI **2** oligomer aggregates are characterized by nearly the same stacking geometries and exciton coupling strengths in the intermediate coupling regime. This parity of intermolecular geometry and intermolecular coupling strength and the disparity of the aggregates length between PBI **1** dimer and PBI **2** oligomer aggregates allow us to conjecture the movement of the coherently delocalized exciton along the PBI **2** oligomer aggregates chain. As the coherent exciton propagates along the aggregates chain, the energetic fluctuations between molecular sites reduce the size of exciton wave function, resulting in exciton localization in PBI **2** oligomer aggregates^9^,^10^. Therefore, exciton transport, inherently inducing exciton localization, is directly reflected in the trace of *N*_coh_ as a fast decay component, while the trace of *I*^0-*ν′*^_(*ν′*>1)_/*I*^0-1^ peak ratio, irrelevant to the exciton delocalization size, remains constant. Consequently, this size-dependent exciton behaviour reveals that the Frenkel exciton generated in self-assemblies consisting of multiple chromophores is initially delocalized over at least three monomeric units and transports rapidly along the aggregates chain in a coherent manner. Since the coherent exciton motion generally ensures fast and efficient energy transfer, our direct experimental observation of exciton delocalization size in extended PBI **2** H-aggregates suggests the possibility of long range energy transfer in more rigid cofacially stacked artificial light-harvesting materials that do not suffer from dynamic disorder and energetic relaxation processes in the excited state. Accordingly, our results stimulate the design of such more sophisticated aggregates, which is prerequisite for optimal device efficiency^3^,^46^,^47^,^48^.

Direct observation of exciton localization in H-aggregates of planar π-conjugated molecules has been accomplished through broadband fluorescence upconversion spectroscopy by means of transient fluorescence from the lower Frenkel state of π-stacked aggregates. Interestingly, at very early times the fluorescence spectra of H-type PBI aggregates exhibit a very similar spectral signature of J-aggregates, where 0-0 vibronic band is dominant. As time evolves, the fluorescence spectra with the clear vibronic progression bands become red-shifted and broad, which are commonly observed for cofacially π-stacked dye ensembles. Thus, by recording the vibronic peak ratio in the transient fluorescence spectra, the initial exciton delocalization size and localization dynamics are directly unravelled. In the simple dimer case, the excimer formation processes and the concomitant decoherence processes occur within several hundreds of femtoseconds. On the other hand, in the extended PBI aggregates, the photoexcitation energy is initially delocalized over at least three molecular units and moves coherently along the chain within tens of femtoseconds, preceding excimer formation process. This comparative study on the smaller PBI dimer and the more device-related extended PBI aggregate stacks suggests the possibility of long range coherent energy transfer in artificial photon-accessing materials. Our current work on the exciton dynamics of PBI aggregates not only paves the way for future experimental and theoretical studies of molecular aggregates but also sets a stage for the use of molecular aggregates in the field of molecular optoelectronic materials.

## Methods

### Femtosecond broadband fluorescence upconversion spectroscopy

A femtosecond broadband fluorescence upconversion apparatus was used for obtaining the transient fluorescence spectra. Detailed information for the femtosecond broadband fluorescence upconversion apparatus is described in Supplementary Methods. A Ti:sapphire laser system (Spectra-Physics, Spitfire) provides 30 fs, 400 μJ pulses at 800 nm with 10 kHz repetition rate. This system drives a non-collinear optical parametric amplifier, from which the visible pump pulse (∼20 fs, ∼3.5 μJ) is generated, and an optical parametric amplifier (TOPAS, Light Conversion), which delivers 50 fs, ∼40 μJ gate pulses in range of 1,150–1,400 nm with the vertical polarization. Both the pump and gate pulses are compressed by using a fused-silica prism pair in visible region and a sequence of SF50 prism pair in near infrared region, respectively. To prevent polarization-dependent signals, the pulse polarization is controlled with a half wave plate to be a magic angle (54.7°) and finally the beam is focused onto a 500-μm-thick quartz cuvette containing sample. The pulse energy is attenuated by the neutral density (ND) filter to keep a level below 60 nJ. Moreover, the cuvette is mounted on a motor-driven stage and continuously moved back and forth to avoid photodegradation and the thermal lens effect. Collection of the fluorescence is achieved by a reflecting microscope objective lens (Newport). Finally, the collected fluorescence is relayed onto the nonlinear crystal by the off-axis parabolic mirror (Newport, focal length: 50 mm). When the collected fluorescence is mixed with the near infrared gate pulse, the horizontally polarized upconverted signal is emitted from the type II nonlinear crystal at an angle different from the original fluorescence. Unwanted light of horizontal polarization, stemming from the original fluorescence and the pump pulse (or Rayleigh scattered light), is mostly ejected by a wire-grid polarizer (Moxtec PPL04C). Moreover, the upconverted signals pass a wire-grid polarizer to eliminate unwanted light of vertical polarization, originating from the remaining original fluorescence. The upconverted signals are imaged dispersion-free onto the entrance slit of a spectrograph (Dongwoo Optron, Monora 320i), and then the upconverted spectrum is finally registered with a CCD camera (Andor Technology, DV420 BU). The full width at half maximum of the cross-correlation functions between the scattered pump pulse (that is, 495 nm) and the gate pulse (that is, 1165, nm) is measured to be 100 fs.

## Additional information

**How to cite this article:** Sung, J. *et al*. Direct observation of ultrafast coherent exciton dynamics in helical π-stacks of self-assembled perylene bisimides. *Nat. Commun.* 6:8646 doi: 10.1038/ncomms9646 (2015).

## Supplementary Material

Supplementary Information Supplementary Figures 1-12, Supplementary Tables 1-3, Supplementary Note 1-3, Supplementary Methods and Supplementary References
