# Supplementary material for: Direct observation of ultrafast coherent exciton dynamics in helical π-stacks of self-assembled perylene bisimides
Source: Nat Commun. 2015 Oct 23;6:8646. doi: 10.1038/ncomms9646 (PMC4639892; doi:10.1038/ncomms9646)
Supplement: Supplementary Information — Supplementary Figures 1-12, Supplementary Tables 1-3, Supplementary Note 1-3, Supplementary Methods and Supplementary References [file ncomms9646-s1.pdf]

Supplementary Figures

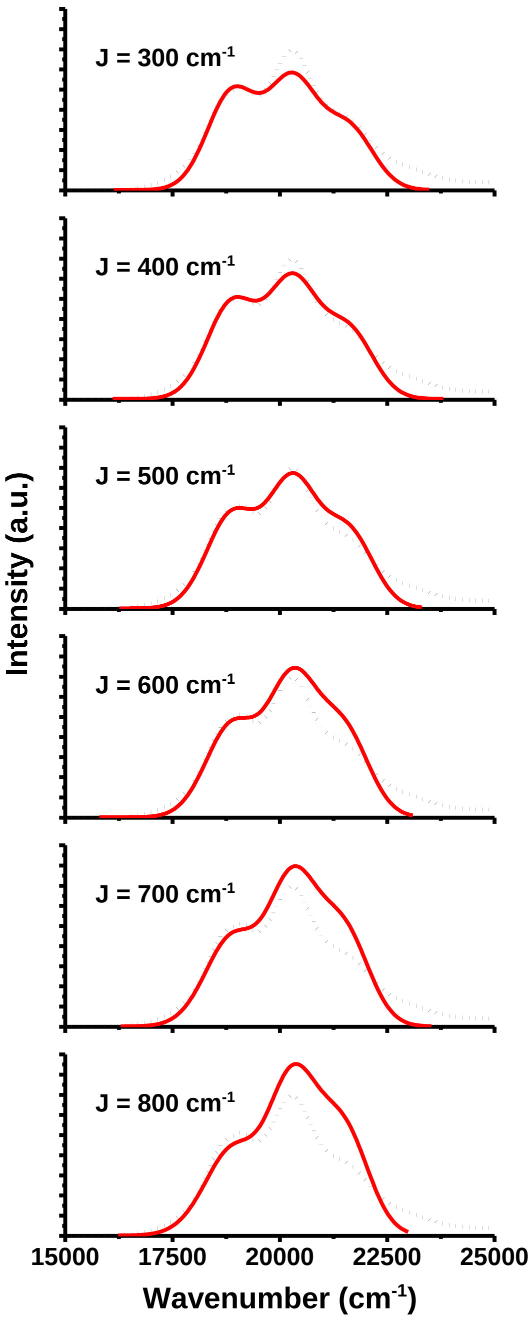

Supplementary Figure 1. Calculated (red solid line) and the measured (grey dotted line) absorption spectra of PBI **1** dimer.

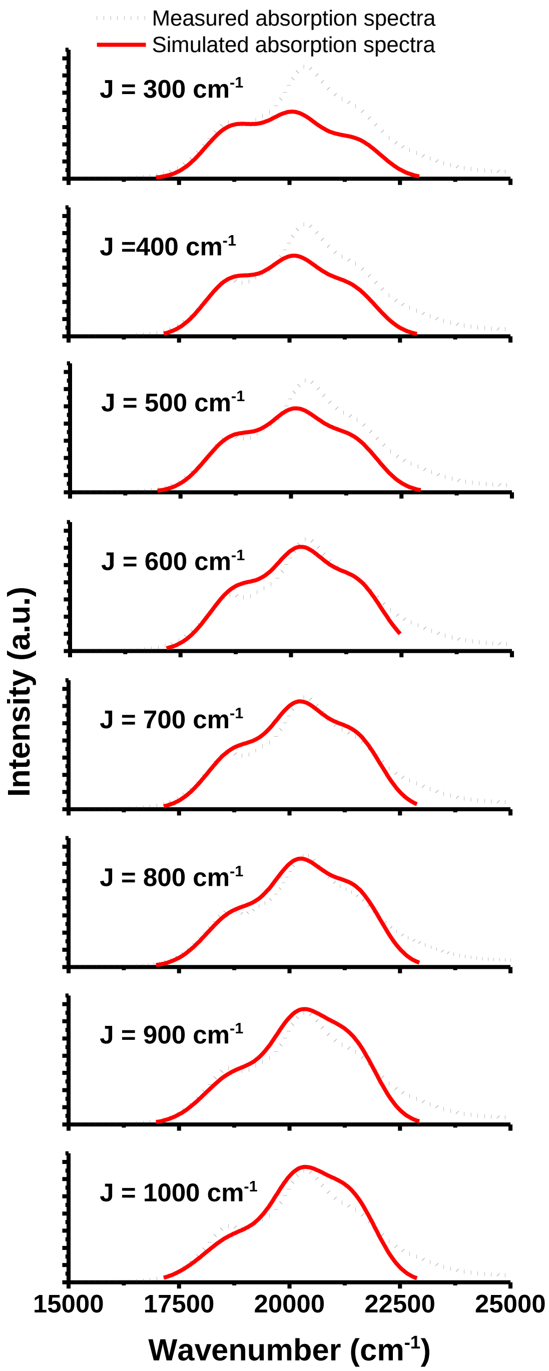

Supplementary Figure 2. Calculated (red solid line) and the measured (grey dotted line) absorption spectra of PBI **2** oligomer aggregates.

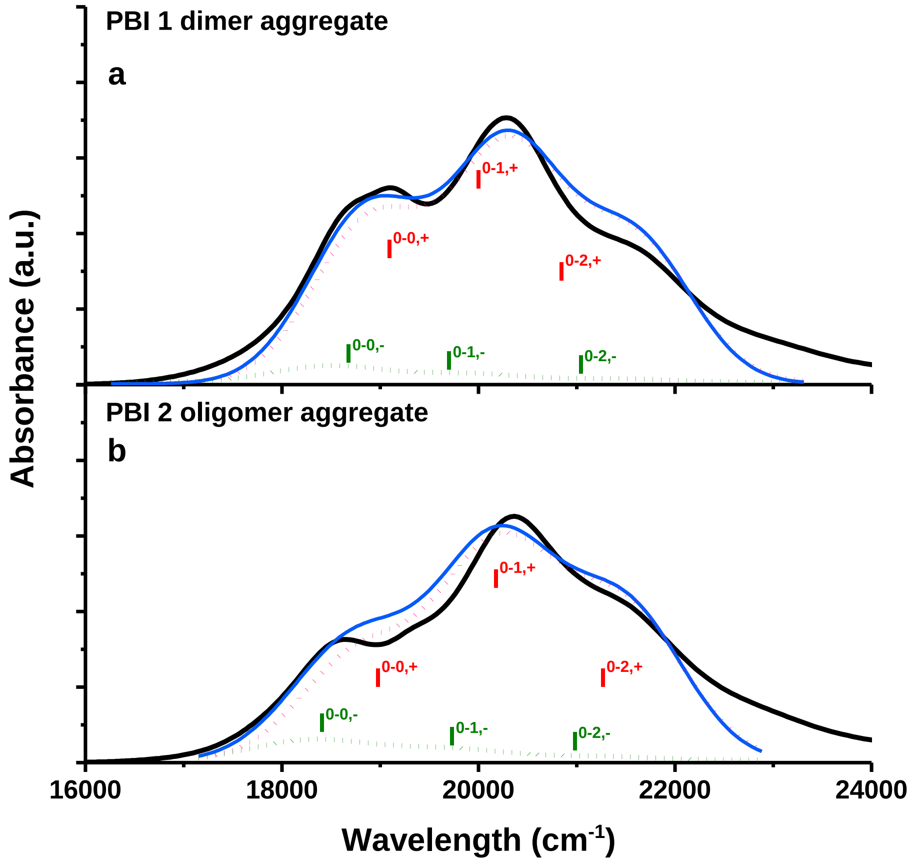

Supplementary Figure 3. Comparison between the measured (black solid line) and best-fit (blue solid line) absorption spectra calculated with the coupling strength of 500 cm^{-1} for PBI **1** dimer (a) and 700 cm^{-1} for PBI **2** oligomer aggregates (b), respectively. Contribution of the symmetric upper band (+) and antisymmetric bottom band (-) are given in red and green dotted line.

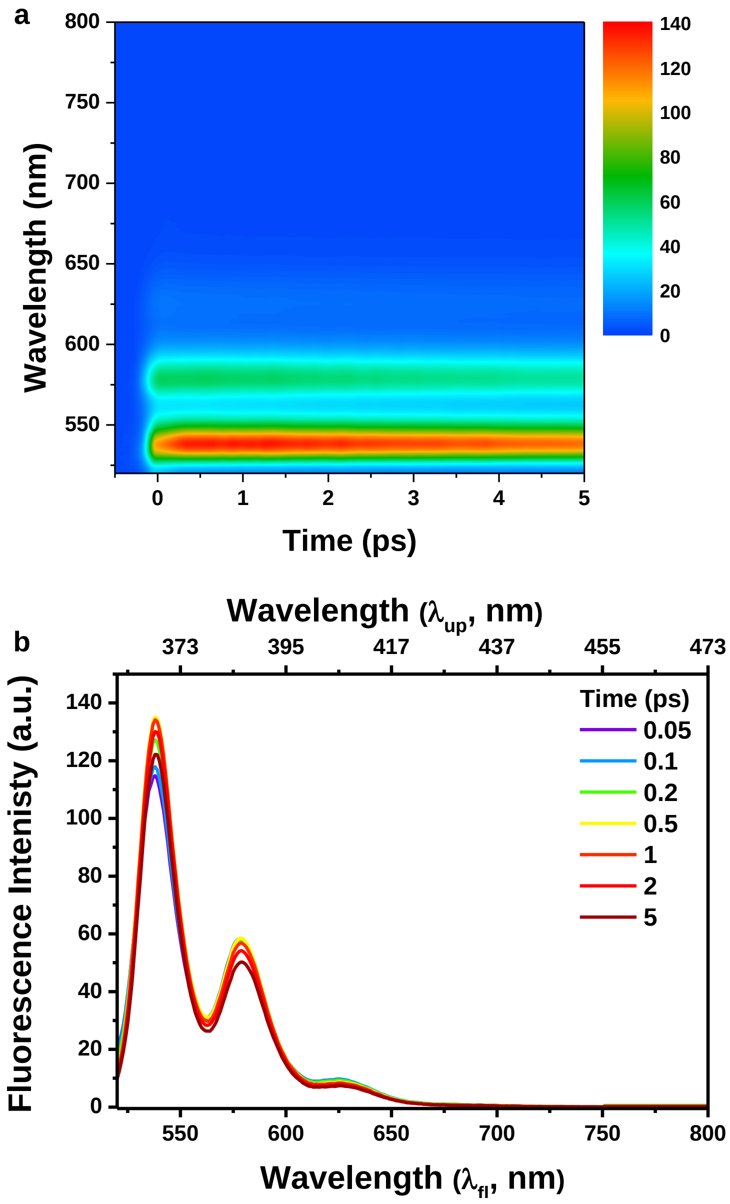

Supplementary Figure 4. (a) 2D fluorescence map and (b) transient fluorescence spectra (bottom panel) of PBI **1** monomer in $CHCl_3$ obtained upon photoexcitation at 495 nm.

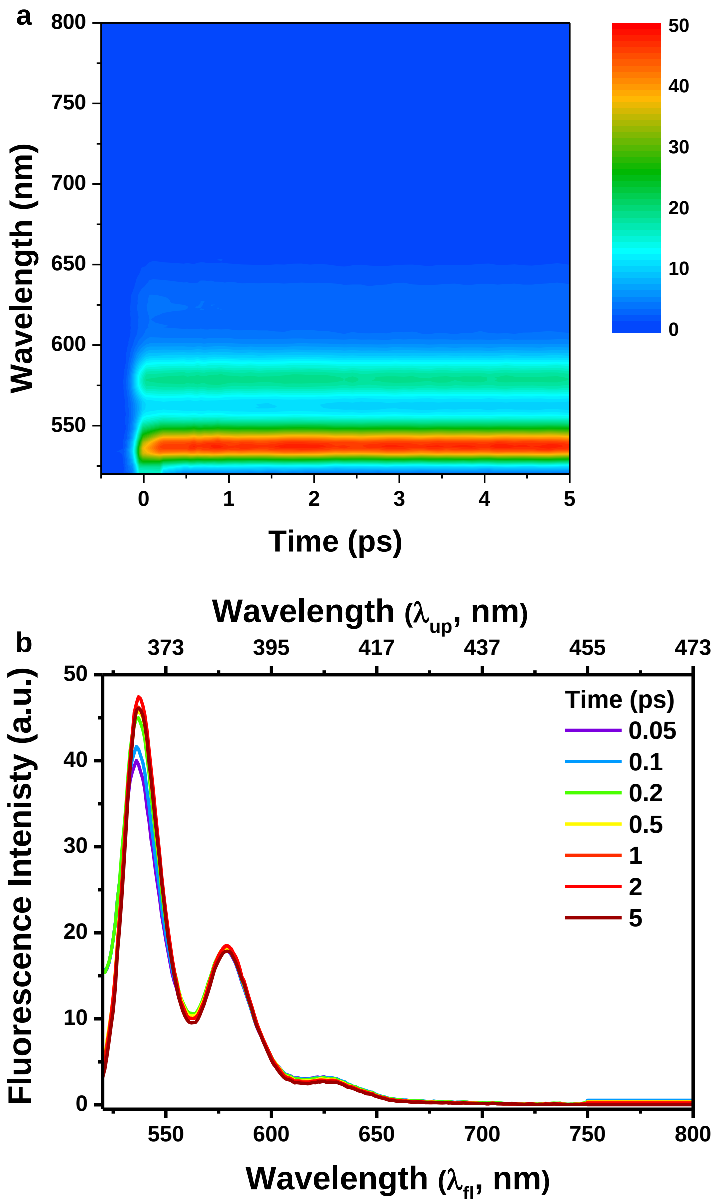

Supplementary Figure 5. (a) 2D fluorescence map and (b) transient fluorescence spectra (bottom panel) of PBI **2** monomer in CH_2Cl_2 obtained upon photoexcitation at 495 nm.

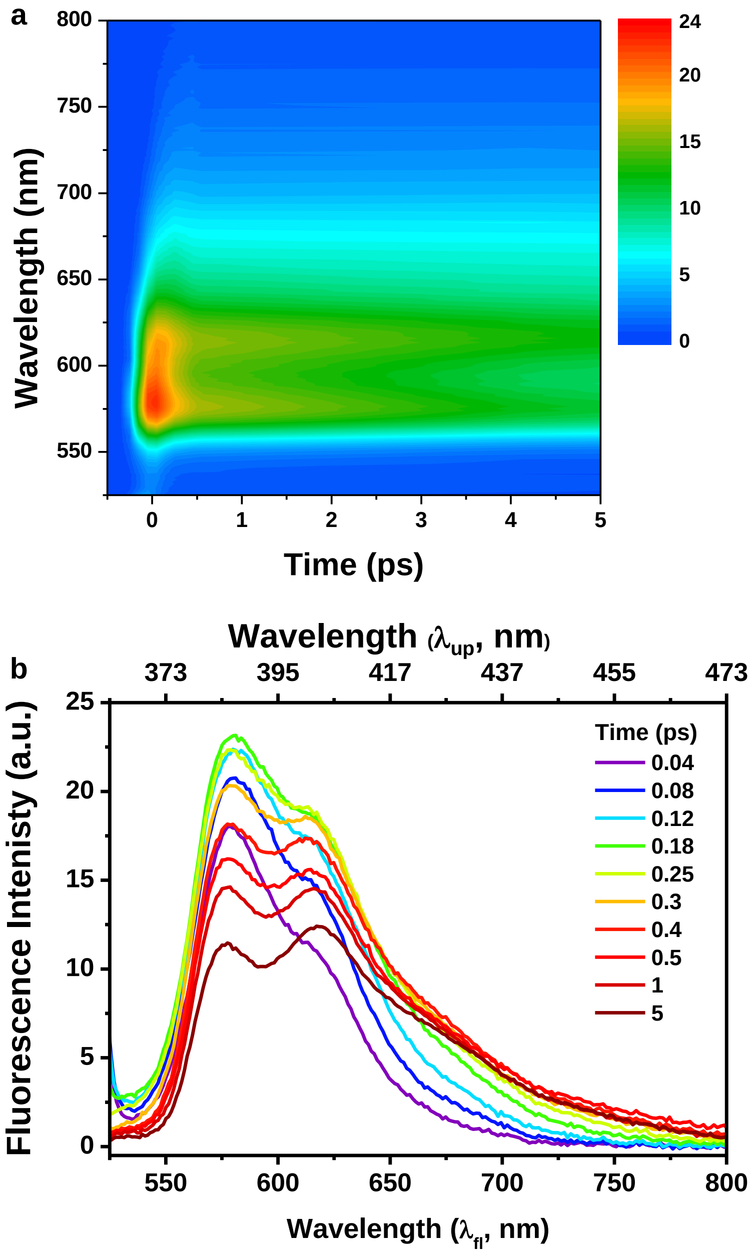

Supplementary Figure 6. (a) 2D fluorescence map and (b) transient fluorescence spectra of PBI **1** dimer (1.0×10^{-2} M) in a solvent mixture of $CHCl_3$/ MCH obtained upon photoexcitation at 495 nm.

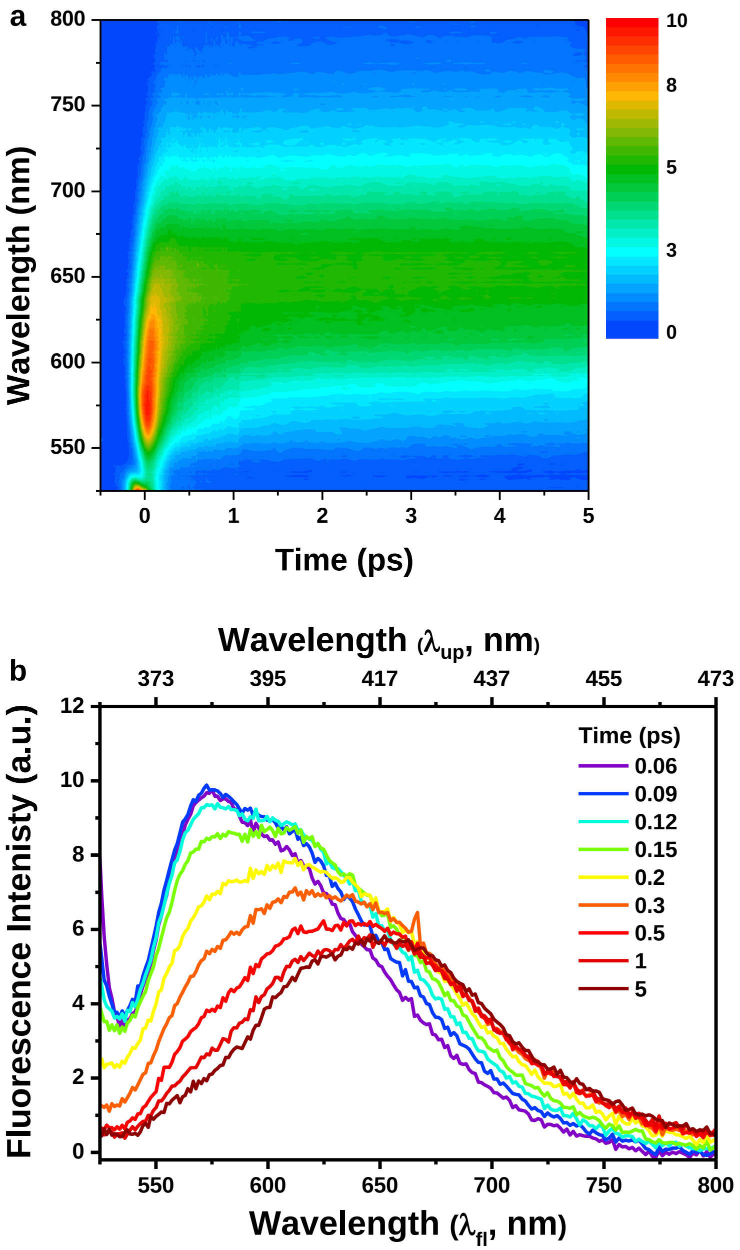

Supplementary Figure 7. (a) 2D fluorescence map and (b) transient fluorescence spectra of PBI **2** oligomer aggregates (1.0×10^{-3} M) in MCH obtained upon photoexcitation at 495 nm.

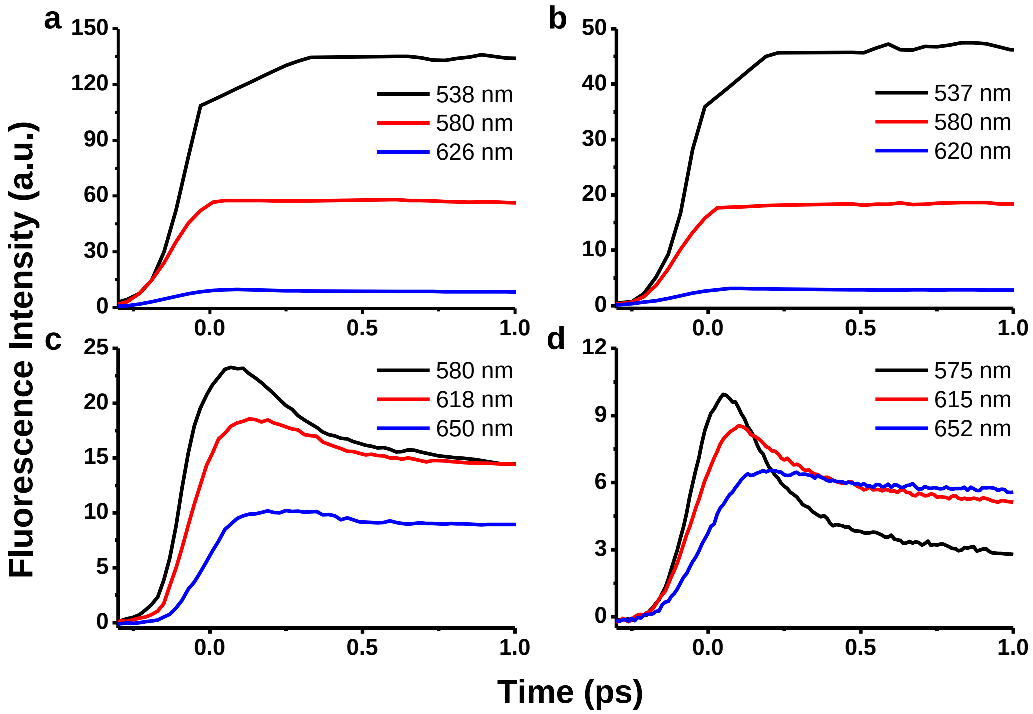

Supplementary Figure 8. Time-resolved fluorescence decay profiles of (a) PBI **1** monomer in $CHCl_3$, (b) PBI **2** monomer in CH_2Cl_2, (c) PBI **1** dimer (1.0×10^{-2} M) in a solvent mixture of $CHCl_3$/ MCH, and (d) PBI **2** oligomer aggregates (1.0×10^{-3} M) in MCH monitored at band maxima of vibronic progression upon photoexcitation at 495 nm.

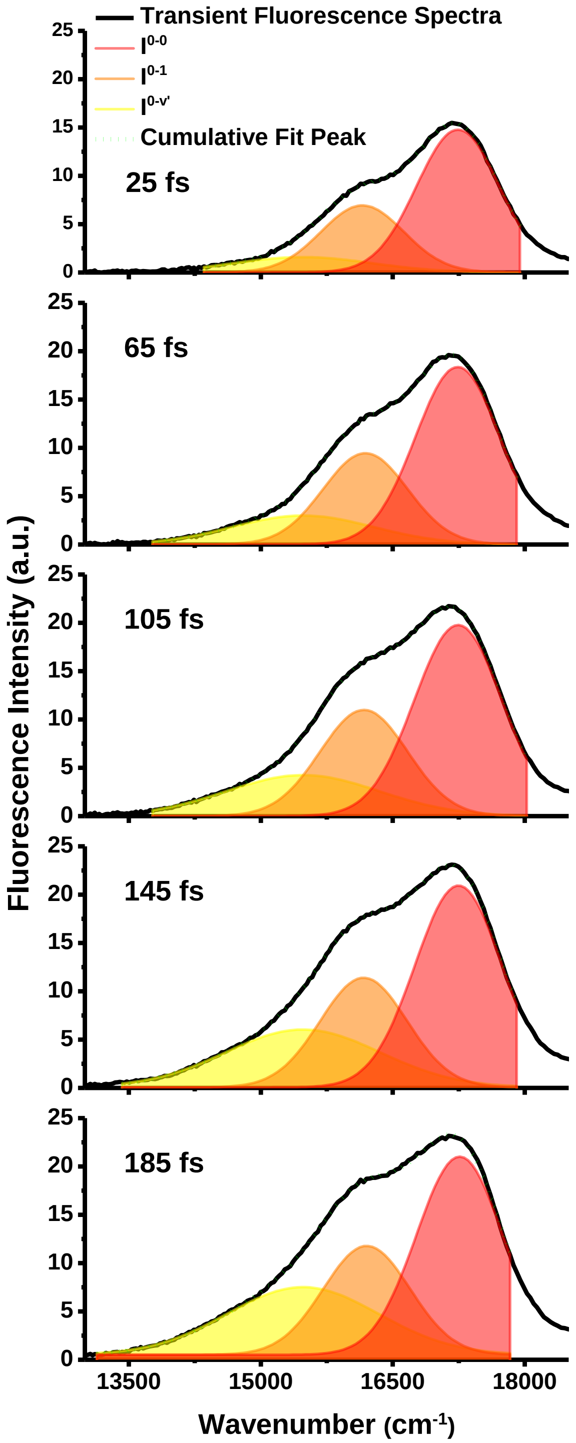

Supplementary Figure 9. Transient fluorescence spectra (black solid line) of PBI **1** dimer ($1.0{\times}10^{-2}$ M) in a solvent mixture of $CHCl_3$/ MCH and simulated fluorescence spectra (green dotted line) plotted with components of a fit to three Gaussian functions.

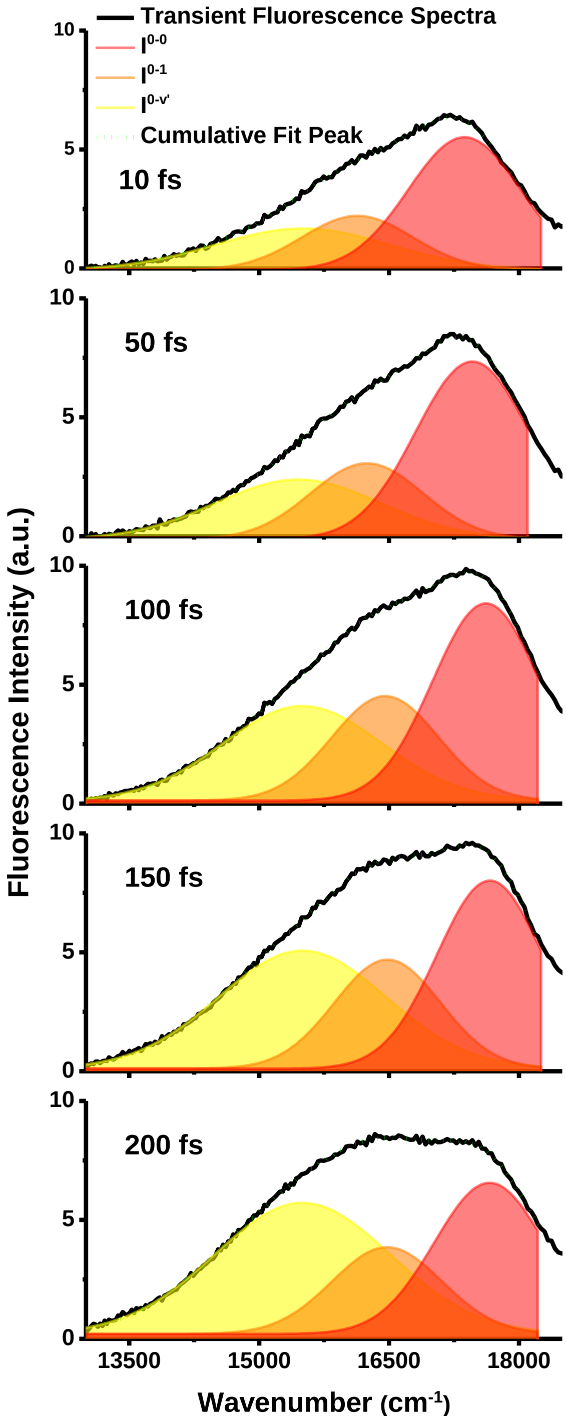

Supplementary Figure 10. Transient fluorescence spectra (black solid line) of PBI **2** oligomer aggregates in MCH (1.0×10^{-3} M) obtained and simulated fluorescence spectra (green dotted line) plotted with components of a fit to three Gaussian functions.

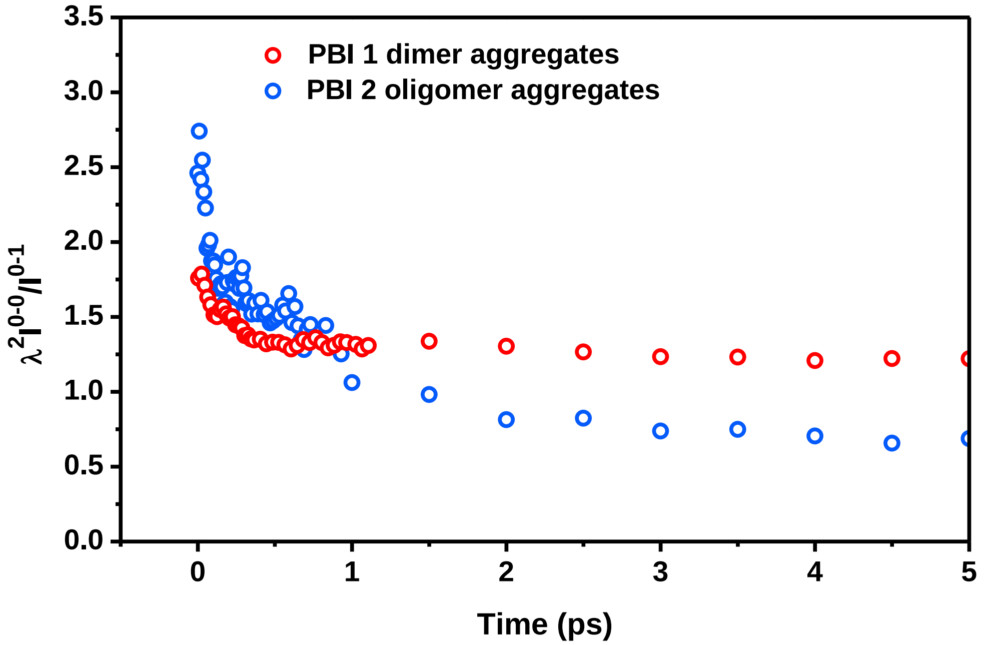

Supplementary Figure 11. The $\lambda^2 I^{0\text{-}0}/ I^{0\text{-}1}$ vibronic peak ratio of PBI **1** dimer (red hollow triangle) and PBI **2** oligomer aggregates (blue hollow circle) as a function of time t^*.

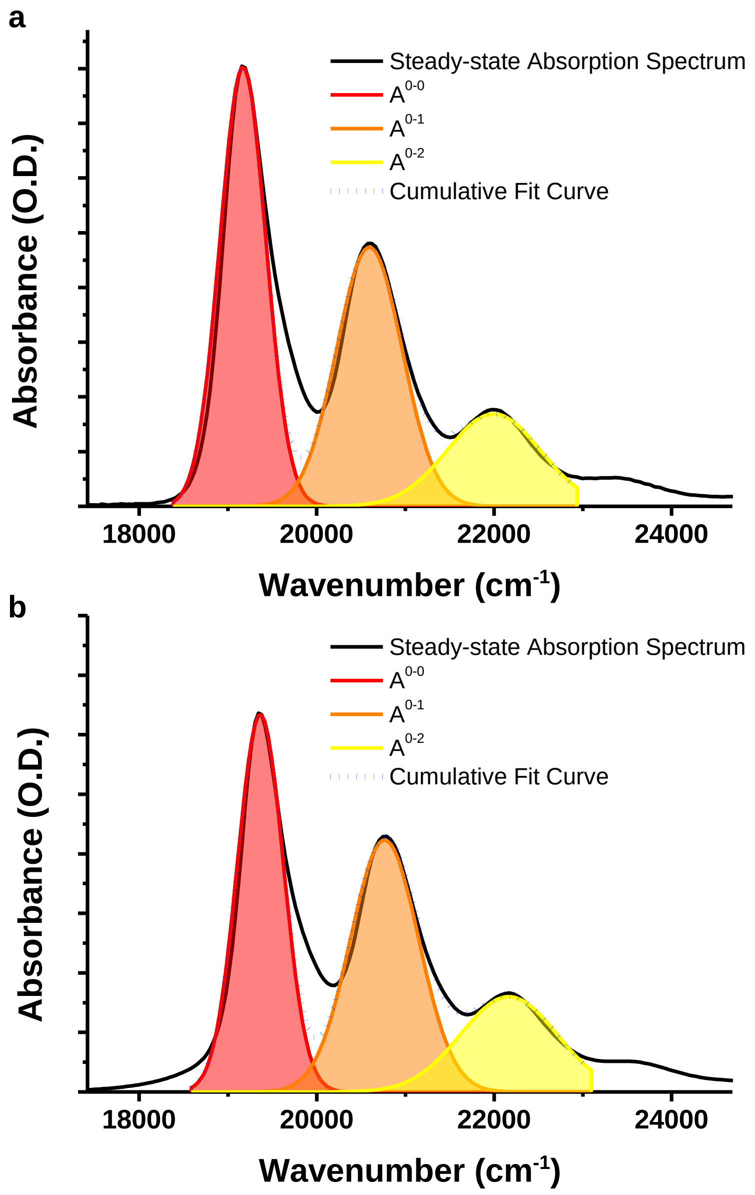

Supplementary Figure 12. Comparison between the measured (black solid line) and best-fit (blue dotted line) absorption spectra for PBI **1** monomer in a solvent mixture of $CHCl_3$/ MCH with 10^{-6} M (a) and PBI **2** monomer in MCH with 10^{-7} M (b), respectively.

Supplementary Tables

		$^{a}x_{c}$ (cm^{-1})	^{b}w(cm^{-1})	^{c}A
	$I^{0\text{-}0}$	17243	940	17400
**25 fs**	$I^{0\text{-}1}$	16156	942	8182
	$I^{0\text{-}v'}$	15480	1567	3102
	$I^{0\text{-}0}$	17240	960	22083
**65 fs**	$I^{0\text{-}1}$	16190	961	11362
	$I^{0\text{-}v'}$	15480	1600	5978
	$I^{0\text{-}0}$	17245	998	24720
**105 fs**	$I^{0\text{-}1}$	16173	997	13723
	$I^{0\text{-}v'}$	15480	1741	9233
	$I^{0\text{-}0}$	17249	990	25904
**145 fs**	$I^{0\text{-}1}$	16170	988	14047
	$I^{0\text{-}v'}$	15480	1755	13156
	$I^{0\text{-}0}$	17262	980	25181
**185 fs**	$I^{0\text{-}1}$	16205	985	13918
	$I^{0\text{-}v'}$	15480	1719	15079

[a]The transition energies, [b]widths and [c]integrated areas of $I^{0\text{-}0}$, $I^{0\text{-}1}$ and $I^{0\text{-}v'}$ vibronic bands.

Supplementary Table 1. Best-fit parameters of transient fluorescence spectra of PBI **1** dimer (1.0×10^{-2} M) in a solvent mixture of $CHCl_3$/ MCH.

		$^{a}x_{c}$ (cm^{-1})	^{b}w(cm^{-1})	^{c}A
	I^{0-0}	17373	1297	9080
**10 fs**	I^{0-1}	16135	1280	3653
	$I^{0-v'}$	15500	2024	4431
	I^{0-0}	17464	1309	12263
**50 fs**	I^{0-1}	16245	1300	5199
	$I^{0-v'}$	15450	1908	6000
	I^{0-0}	17622	1237	12869
**100 fs**	I^{0-1}	16457	1235	6799
	$I^{0-v'}$	15500	1839	9172
	I^{0-0}	17666	1223	12125
**150 fs**	I^{0-1}	16483	1225	7033
	$I^{0-v'}$	15500	1938	12050
	I^{0-0}	17662	1275	10159
**200 fs**	I^{0-1}	16478	1273	5817
	$I^{0-v'}$	15500	2027	14009

^{a}The transition energies, ^{b}widths and ^{c}integrated areas of I^{0-0}, I^{0-1} and $I^{0-v'}$ vibronic bands.

Supplementary Table 2. Best-fit parameters of transient fluorescence spectra of PBI **2** oligomer aggregates in MCH (1.0×10^{-3} M)

		$^{a}x_c$ (cm^{-1})	^{b}w(cm^{-1})	^{c}A
PBI **1**	$\boldsymbol{A^{0-0}}$	19168	519	1045
	$\boldsymbol{A^{0-1}}$	20592	740	879
	$\boldsymbol{A^{0-2}}$	21995	1051	445
PBI **2**	$\boldsymbol{A^{0-0}}$	19365	517	2057
	$\boldsymbol{A^{0-1}}$	20764	770	2042
	$\boldsymbol{A^{0-2}}$	22165	1084	1087

[a]The transition energies, [b]widths and [c]integrated areas of A^{0-0}, A^{0-1} and A^{0-2} vibronic bands.

Supplementary Table 3. Best-fit parameters of transient absorption spectra of PBI **1** dimer (1.0×10^{-2} M) in a solvent mixture of $CHCl_3$/ MCH with 10^{-6} M and PBI **2** monomer in MCH with 10^{-7} M, respectively.

Supplementary Note

Supplementary Note 1: Determination of the coupling strength. The excitonic coupling strength of PBI **1** dimer and PBI **2** oligomer aggregates were identified by comparison of the measured absorption spectra with the theoretical model. According to Knapp, in a weak coupling region, the single-particle approximation (SPA) absorption spectrum for the excitons in bands with υ_t *P* 0 can be expressed as follows[4-6],

$$A_{SPA}(\omega) \approx (N\mu^2)^{-1} \sum_{\upsilon_t, P, 0} F_{G,\upsilon_t} \frac{1}{\sqrt{\pi}\sigma_{MN}} e^{-(\omega-\omega^{(2)SPA}_{k=0,\upsilon_t})^2/\sigma^2_{MN}} \tag{1}$$

F_{G,υ_t} is the oscillator strength of the state $\mathbf{k} = 0,\upsilon_t$ exciton and is evaluated under the single-particle approximation (SPA) as

$$F_{G,\upsilon_t} \approx Nf^2_{0,\tilde{\upsilon}=\upsilon_t}\mu^2 \left(1 - \frac{We^{-\lambda^2}}{2\omega_0} G(\upsilon_t;\lambda^2)\right)^2 \tag{2}$$

with the single molecule $0,\upsilon_t$ oscillator strength, $f^2_{0,\tilde{\upsilon}=\upsilon_t}\mu^2$, the free exciton bandwidth, *W*, and the function, $G(\upsilon_t;\lambda^2) \equiv \sum_{\upsilon(\neq\upsilon_t)} \frac{\lambda^{2\upsilon}}{\upsilon!(\upsilon-\upsilon_t)}$. Accordingly, a general expression of the vibronic peak ratios for any aggregates takes forms of

$$R \equiv \frac{A_{\upsilon_t,1}}{A_{\upsilon_t,2}} \equiv \frac{F_{G,\upsilon_t,1}}{F_{G,\upsilon_t,2}} = \left(\frac{f_{G,\upsilon_t,1}\mu(1\text{-}G(\upsilon_{t,2};\lambda^2)e^{-\lambda^2}W/2)}{f_{G,\upsilon_t,2}\mu(1\text{-}G(\upsilon_{t,2};\lambda^2)e^{-\lambda^2}W/2)}\right)^2 = \frac{I^{0\text{-}\upsilon_t,1}}{I^{0\text{-}\upsilon_t,1}} \tag{3}$$

Moreover, when taking into account the symmetry (+J) and antisymmetry (-J) components of spectrum, such expression can be rewritten as follows[2],

$$\left(\frac{f_{0,\upsilon_t,1}\mu(1-G(\upsilon_{t,2};\lambda^2)e^{-\lambda^2}J_{\pm}/\omega_0)}{f_{0,\upsilon_t,2}\mu(1-G(\upsilon_{t,2};\lambda^2)e^{-\lambda^2}J_{\mp}/\omega_0)}\right)^2=\frac{I^{(0\text{-}\upsilon_t,1)^{\pm}}}{I^{(0\text{-}\upsilon_t,1)^{\mp}}} \tag{4}$$

Such expression can be directly applied to the H-aggregates and therefore, enables us to simulate the absorption spectra of PBI **1** dimer and PBI **2** oligomer aggregates.

For PBI **1** dimer, the 0-0 to 0-1 and 0-1 to 0-2 vibronic peak ratios are derived as below,

$$\frac{I^{(0\text{-}0)^{\pm}}}{I^{(0\text{-}1)\pm}}=\frac{1}{0.84}\left(\frac{(1\mp 0.456e^{-\lambda^2}J_{\pm}/\omega_0)}{(1\pm 0.255e^{-\lambda^2}J_{\mp}/\omega_0)}\right)^2 \tag{5}$$

$$\frac{I^{(0\text{-}1)^{\pm}}}{I^{(0\text{-}2)^{\pm}}}=\frac{1}{0.51}\left(\frac{(1\mp 0.255e^{-\lambda^2}J_{\pm}/\omega_0)}{(1\pm 0.5316e^{-\lambda^2}J_{\mp}/\omega_0)}\right)^2 \tag{6}$$

For PBI **2** oligomer aggregates, the 0-0 to 0-1 and 0-1 to 0-2 vibronic peak ratios are expressed as below,

$$\frac{I^{(0\text{-}0)^{\pm}}}{I^{(0\text{-}1)\pm}}=\frac{1}{0.99}\left(\frac{(1\mp 0.483e^{-\lambda^2}J_{\pm}/\omega_0)}{(1\pm 0.129e^{-\lambda^2}J_{\mp}/\omega_0)}\right)^2 \tag{7}$$

$$\frac{I^{(0\text{-}1)^{\pm}}}{I^{(0\text{-}2)^{\pm}}}=\frac{1}{0.52}\left(\frac{(1\mp 0.129e^{-\lambda^2}J_{\pm}/\omega_0)}{(1\pm 0.482e^{-\lambda^2}J_{\mp}/\omega_0)}\right)^2 \tag{8}$$

Additionally, the relative intensity of symmetric (+) and antisymmetric (-) components is given by (1-cos(180-β))/ (1+cos(180-β)), where β is the angle between the neighboring monomer transition dipoles[7,8]. By applying this relationship between vibronic peaks, we

have successively simulated the absorption spectra of PBI **1** dimer and PBI **2** oligomer aggregates by changing exciton coupling strength. We used a Gaussian line shape with a full width at half-maximum of ω_0. The resultant absorption spectra calculated for PBI **1** dimer and PBI **2** oligomer aggregates with various exciton coupling strengths are shown in Supplementary Figs 1, 2 and 3. By comparison of the simulated absorption spectra with the measured absorption spectra, we have estimated the exciton coupling strength of ~ 500 cm^{-1} for PBI **1** dimer and that of ~ 700 cm^{-1} for PBI **2** oligomer aggregates.

Supplementary Note 2: Determination of the Huang-Rhys factor (λ^2). The Huang-Rhys (HR) factor is used to characterize vibronic coupling. In principal, the HR factor is related to the displacement between the minimum energy positions between the ground and excited harmonic vibrational potential surface by the expression, λ^2 = $\Delta^2/2$. The nuclear overlap integral can be well-reproduced by using the definition of the Gaussian forms of the wave functions and therefore, can be expressed as $S_{0,0} = e^{-\lambda^2/2}$, $S_{0,1} = \sqrt{\lambda^2} e^{-\lambda^2/2}$, and etc. Since the Franck-Condon (FC) overlap factors are the square of the nuclear overlap integral between the ground and excited state, the FC intensities in the single molecule spectrum are determined through the Poissonian distribution, $I^{0\text{-}v} = \frac{\lambda^{2v} e^{-\lambda^2}}{v!}$. As determined from the relationship, the relative intensity ratio of the 0-1 to the 0-0 absorption band becomes the value of λ^2. By using this relationship,

we could obtain the HR factors for PBI **1** and PBI **2** from the absorption spectra of their corresponding PBI monomers in solution. The steady-state absorption spectra of PBI **1** in a solvent mixture of $CHCl_3$/MCH with 10^{-6} M and PBI **2** in MCH with 10^{-7} M were well-fitted with the sum of three Gaussians. The resultant fits are shown in Supplementary Figure 12 and several representative parameters extracted from these fits are listed in Supplementary Table 3. As a consequence, we obtained the HR factors of 0.84 for PBI **1** and 0.99 for PBI **2** from the absorption spectra of their corresponding PBI monomers in solution.

Supplementary Note 3: Determination of the N_{coh}. We have adopted here Spano's explanation to quantify the delocalization of excitation[9-12]. For the sake of simplicity, we would give an overview of the derivation process for the coherence length. The coherence length is derived based on the coherence function, which is given by

$$C^{(em)} \equiv \left\langle \psi^{(em)} \middle| B_{\mathrm{n}}^{+} B_{\mathrm{n+s}} \middle| \psi^{(em)} \right\rangle \tag{9}$$

where the $\mathbf{B}^{+}$ is the local exciton creation operator. The time-dependent coherence field is given by

$$C(\mathrm{t},\mathrm{r}) \equiv \mathrm{Tr}(\hat{\mathbf{C}}(\mathrm{r})\rho^{(2)}(t,0)) \tag{10}$$

where the $\hat{\mathbf{C}}(\mathrm{r})$ is the coherence field operator and the $\rho^{(2)}(t_2,t_1)$ is an excited state, resulting from the two light interactions. Derived from the interaction of the emitting exciton with this coherence field, the coherence length is defined as

$$N_{\mathrm{coh}}(t,r) \equiv \frac{1}{\left\langle C^{(em)}(0)\right\rangle} \sum_{\mathrm{s}} \left| \left\langle C^{(em)}(\mathrm{s}) \right\rangle_{\mathrm{C}} \right| \tag{11}$$

This equation can be rewritten using the relationship between the relative ratio of vibronic bands and the coherence length as shown below,

$$N_{\mathrm{coh}} \approx \lambda^2 \frac{\left\langle I^{0-0} \right\rangle_{\mathrm{C}}}{\left\langle I^{0-1} \right\rangle_{\mathrm{C}}} \tag{12}$$

To track down variation in the coherence length, we have obtained the change in the relative intensity of the vibronic bands of transient fluorescence spectra. Followed by a Frank-Condon (FC) model, the vibronic transitions of fluorescence spectrum can be described by[13]

$$I_{0\to\upsilon} \propto \left(h\omega\right)^3 \mathrm{n}_f^3 \frac{\lambda^{2\upsilon}\exp(-\lambda^2)}{\upsilon!} \tag{13}$$

where n_f is the real part of the refractive index at photon energy $\hbar\omega$. λ^2 and m denote the HR factor and the vibrational level, respectively. An iterative method for fitting the FC model to transient fluorescence spectrum is applied here. We have used a Gaussian as the line shape function for each of the vibronic replicas[13,14]. As shown in Supplementary Figs 9 and 10, the transient fluorescence spectra were well-fitted with the sum of three

Gaussians. The transition energies of $I^{0\text{-}0}$, $I^{0\text{-}1}$ and $I^{0\text{-}v'}$ vibronic bands were initially set at 17400, 16000 and 15000 cm^{-1}, respectively, and then allowed to fluctuate freely for the best fits. Their amplitudes were also allowed to fluctuate freely for the best fits. While the widths of $I^{0\text{-}0}$ and $s^{0\text{-}1}$ bands were set to converge into the similar values, that of $I^{0\text{-}v'}$ was set to an independently adjustable parameter. The resultant fits are shown in Supplementary Figs 9 and 10, and several representative parameters extracted from these fits are listed in Supplementary Tables 1 and 2.

Supplementary Methods

Steady-state absorption and fluorescence spectra. Absorption spectra were obtained by using UV-VIS-NIR spectrometer (Varian, Cary5000), steady-state fluorescence spectra were measured by a Hitachi model F-2500 fluorescence spectrophotometer. Spectral sensitivity was corrected with the comparison of the well-known choromophores such as rhodamine and coumarin dyes.

Femtosecond broadband fluorescence upconversion spectroscopy. A femtosecond broadband fluorescence up-conversion apparatus was used for obtaining the transient fluorescence spectra. A Ti:sapphire laser system (Spectra-Physics, Spitfire) provides 30 fs, 400 μJ pulses at 800 nm with 10 kHz repetition rate. The output beam is divided by beam splitter with the equivalent ratio. A pulse of 200 μJ is used to pump an optical parametric amplifier (TOPAS, Light Conversion) which delivers 50 fs, ~40 μJ gate pulses in range of 1150-1400 nm with the vertical polarization. For achieving the best experimental condition, the wavelength of gates pulse was selected from 1160 to 1450 nm depending on the wavelengths of pump pulse and fluorescence. The gate beam passes through a periscope, which adjusts the height and rotates the vertically polarized pulse to be horizontally polarized. Then, the gate pulse passes a sequence of SF50 prism (55.5°) compressor with optimal separation of 12 cm between the apexes of each prism. Finally, the gate beam is relayed onto the nonlinear crystal (Eksma, type II BBO, θ 32 °,

ϕ 0°, d 1 mm) by a lens (*f* 100 mm Tc 2 mm). The pulse energy of the gate beam is attenuated by the ND filter to keep a level below 50 mW. The rest of the fundamental light is used as a source for the tunable homemade optical parametric amplifiers (OPA) system. This homemade OPA system is based on noncollinearly phase-matching geometry which is easily color-tuned by controlling optical decay between white light continuum seed pulses (450-1400 nm) produced by using a sapphire window (d 2 mm) and visible pump pulses (400 nm) generated by frequency doubling nonlinear crystal (Eksma, type I BBO). The generated visible OPA pulses had a pulse width of ~20 fs and an average power of 35 mW at 10 kHz repetition rate in the range of 480-700 nm with vertical polarization. In order to adjust the pulse polarization to prism compressors, the pulse passes through the periscope and then passes through a fused-silica prism compressor (69°), which has the optimal separation of 95 cm. In order to prevent polarization-dependent signals, the pulse polarization is controlled with a half wave plate to be a magic angle (54.7°) and finally the beam is focused onto a 500 μm thick quartz cuvette containing sample with a lens (*f* 300 mm, Tc 2 mm). The pulse energy is attenuated by the ND filter to keep a level below 60 nJ. Moreover, the cuvette is mounted on a motor-driven stage and continuously moved back and forth to avoid photo-degradation and the thermal lens effect. Collection of the fluorescence is achieved by a reflecting microscope objective lens (Newport). Finally, the collected fluorescence is relayed onto the nonlinear crystal by the off-axis parabolic mirror (Newport, *f* 50 mm).

The horizontally polarized upconverted signal is emitted from the type II nonlinear crystal at an angle different from the original fluorescence. Unwanted light of horizontal polarization, stemming from the original fluorescence and the pump pulse (or Rayleigh scattered light), is mostly ejected by a wire-grid polarizer (Moxtec PPL04C). Moreover, the upconverted signals pass a wire-grid polarizer (Moxtec PPL04C) in order to eliminate unwanted light of vertical polarization, originating from the remaining original fluorescence. The upconverted signals are imaged dispersion-free onto the entrance slit of a spectrograph (Dongwoo Optron, Monora 320i) and then the upconverted spectrum is finally registered with a CCD camera (Andor Technology, DV420 BU). The FWHM of the cross-correlation functions between the scattered pump pulse (i.e., 495 nm) and the gate pulse (i.e., 1165 nm) is measured to be 100 fs.

Supplementary References

(1) Shreve, A. P., Haroz, E. H., Bachilo, S. M., Weisman, R. B., Tretiak, S., Kilina, S. & Doorn, S. K. Determination of exciton-phonon coupling elements in single-walled carbon nanotubes by Raman overtone analysis. *Phys. Rev. Lett.* **98**, 037405 (2007).

(2) Kistler, K. A., Pochas, C. M., Yamagata, H., Matsika, S. & Spano, F. C. Absorption, circular dichroism, and photoluminescence in perlyene diimide bichromophores: Polarization-dependent H- and J-aggregate behavior. *J. Phys. Chem. B* **116**, 77-86 (2012).

(3) Ho, P. K. H., Kim, J.-S., Tessler, N. & Friend, R. H. Photoluminescence of poly(*p*-phenylenevinylene)-silica nanocom posites: Evidence for dual emission by Franck–Condon analysis. *J. Chem. Phys.* **115**, 2709-2720 (2001).

(4) Spano, F. C. Absorption in region-regular poly(3-hexyl)thiophene thin films: Fermi resonances, interband coupling and disorder. *Chem. Phys.* **325**, 22-35 (2006).

(5) Knapp, E. W. Lineshapes of molecular aggregates, exchange narrowing and intersite correlation. *Chem. Phys.* **85**, 73-82 (1984).

(6) Spano, F. C. Modeling disorder in polymer aggregates: The optical spectroscopy of regioregular poly(3-hexylthiophene) thin films. *J. Chem. Phys.* **122**, 234701 (2005).

(7) Seibt, J., Marquetand, P., Engel, V., Chen, Z., Dehm, V. & Würthner, F. On the geometry dependence of molecular dimer spectra with an application to aggregates of perylene bisimide. *Chem. Phys.* **328**, 354-362 (2006).

(8) Guthmuller, J., Zutterman, F. & Champagne, B. Multimode simulation of dimer absorption spectra from first principles calculations: Application to the 3,4,9,10-perylenetetracarboxylic diimide dimer. *J. Chem. Phys.* **131**, 154302 (2009).

(9) Spano, F.C., Clark, J., Silva, C. & Friend, R. H. Determining exciton coherence from the photoluminescence spectral line shape in poly(3-hexylthiophene) thin films. *J. Chem. Phys.* **130**, 074904 (2009).

(10) Spano, F. C. Meskers. S. C. J., Hennebicq, E. & Beljonne, D. Probing excitation delocalization in supramolecular chiral stacks by means of circularly polarized light: Experiment and modeling. *J. Am. Chem. Soc.* **129**, 7044-7054 (2007).

(11) Spano, F. C. & Yamagata, H. Vibronic coupling in J-aggregates and beyond: A direct means of determining the exciton coherence length from the photoluminescence spectrum. *J. Phys. Chem. B* **115**, 5133-5143 (2011).

(12) Tempelaar, R., Spano, F. C., Knoester, J. & Jansen, T. L. C. Mapping the evolution of spatial exciton coherence through time-resolved fluorescence. *J. Phys. Chem. Lett.* **5**, 1505-1510 (2014).

(13) Clark, J., Silva, C., Friend, F. H. & Spano, F. C. Role of intermolecular coupling in the photophysics of disordered organic semiconductors: Aggregate emission in regioregular polythiophene. *Phys. Rev. Lett.* **98**, 206406 (2007).

(14) Chang, M. –H., Hoffmann, M., Anderson, H. L. & Herz, L. M. Dynamics of excited-state conformational relaxation and electronic delocalization in conjugated porphyrin oligomers. *J. Am. Chem. Soc.* **130**, 10171-10178 (2008).
